# Nutmeg Beyond Spice: A Review on Its Therapeutic Potential, Safety and Industrial Promise

**DOI:** 10.1002/fsn3.71053

**Published:** 2025-10-14

**Authors:** Duaa Tariq, Muhammad Tauseef Sultan, Ahmad Mujtaba Noman, Hassan Raza, Shazia Akram, Hagar M. Mohamed, Muhammad Atif Khan, Muhammad Imran, Muzzamal Hussain, Anjuman Gul Memon, Muhammad Atif, Gamal A. Mohamed, Sabrin R. M. Ibrahim, Entessar Al Jbawi

**Affiliations:** ^1^ Department of Human Nutrition, Faculty of Food Science and Nutrition Bahauddin Zakariya University Multan Pakistan; ^2^ Department of Food Science and Technology, Faculty of Food Science and Nutrition Bahauddin Zakariya University Multan Pakistan; ^3^ Department of Medical Laboratory Analysis, College of Medical and Health Sciences Liwa University Abu Dhabi United Arab Emirates; ^4^ Department of Applied Medical Chemistry, Medical Research Institute Alexandria University Alexandria Egypt; ^5^ Department of Human Nutrition and Dietetics The University of Faisalabad Faisalabad Pakistan; ^6^ Department of Food Science and Technology University of Narowal Narowal Pakistan; ^7^ Department of Food Science Government College University Faisalabad Faisalabad Pakistan; ^8^ Department of Biochemistry, College of Medicine Qassim University Buraydah Saudi Arabia; ^9^ Department of Clinical Laboratory Sciences, College of Applied Medical Sciences Jouf University Sakaka Saudi Arabia; ^10^ Department of Natural Products and Alternative Medicine, Faculty of Pharmacy King Abdulaziz University Jeddah Saudi Arabia; ^11^ Department of Chemistry, Preparatory Year Program Batterjee Medical College Jeddah Saudi Arabia; ^12^ Sugar Beet Research Department, Crop Research Administration General Commission for Scientific Agricultural Research (GCSAR) Damascus Syria

**Keywords:** health benefits, industrial application, *myristica fragrans*, phytochemistry, toxicity

## Abstract

With the advancement of scientific knowledge and global awareness, a trend of utilizing natural resources, such as herbal and medicinal plants, for disease prevention and treatment. *Myristica fragrans* from the Myristicaceae family offers various medicinal benefits. It is widely used as a culinary spice and has a long history in traditional medicine for its various therapeutic applications. This review highlights the current findings on the pharmacological potential of *Myristica fragrans* , focusing on its health benefits and applications. Scientific literature was explored using the ScienceDirect, Google Scholar, and PubMed databases. The studies reported that it contains alkaloids, terpenoids, phenols, flavonoids, and glycosides, with myristicin, macelignan, safrole, and sabinene as prime bioactive components. *M. fragrans* exhibits antioxidant, anti‐inflammatory, antidiabetic, antimicrobial, and anticancer properties by modulating various pathways like PI3K/Akt/mTOR, MAPK, and NF‐κB signaling Pathways and G0/G1 or G2/M phase arrest. Moreover, other compounds such as dehydrodiisoeugenol, malabaricone B and C, elemicins, have also shown strong antioxidant potential and enzyme inhibitory properties, which enhance insulin sensitivity, inhibit α‐glucosidase, reduce oxidative stress, and support neurocognitive function by inhibiting inflammatory cytokines (IL‐6, IL‐1β, and TNF‐α). At high doses, gastrointestinal and hepato‐renal adverse effects have been reported. However, with careful usage, it presents significant therapeutic potential. This article also focuses on industrial applications of *M. fragrans* as a medicine, a cosmetic agent, and in food for its aroma and functional properties. However, more clinical research is required to validate these findings and optimize effective and safe use in clinical applications.

## Graphical abstract

This review highlights the current findings on the pharmacological potential of *M. fragrans* , focusing on its health benefits and applications. *M. fragrans* contains alkaloids, terpenoids, phenols, flavonoids and glycosides; with myristicin, macelignan, safrole and sabinene as prime bioactive components. It exhibits antioxidant, anti‐inflammatory, antidiabetic, antimicrobial and anticancer properties. It has significant therapeutic potential. This article also focuses on industrial applications of *M. fragrans* as a medicine, a cosmetic agent, and in food for its aroma and functional properties.

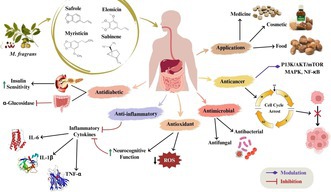

**Abbreviations**
AChE: Acetylcholinesterase
ADP: Adenosine Diphosphate
AEN: Aqueous Extract of Nutmeg
AMPK: AMP‐Activated Protein Kinase
BBB: Blood–Brain Barrier
BChE: Butyrylcholinesterase
Bcl‐2: B‐cell lymphoma 2
BIC: Bicuculline
CAT: Catalase
COX: Cyclooxygenase
DHGA: Dihydroxy Gymnemic Acid
DPPH: 2,2‐diphenyl‐1‐picrylhydrazyl
FAAH: Fatty Acid Amide Hydrolase
FASN: fatty acid synthase
GABA: Gamma‐Aminobutyric Acid
GAE: Gallic Acid Equivalent
GC–MS: Gas Chromatography–Mass Spectrometry
GSH‐Px: Glutathione Peroxidase
HPLC: High‐Performance Liquid Chromatography
IC50: Half‐Maximal Inhibitory Concentration
LDH: Lactate Dehydrogenase
LNX: Lignan‐Enriched Nutmeg
LPO: Lipid Peroxidation
LPS: Lipopolysaccharide‐
MAPK: Mitogen‐Activated Protein Kinase MDA: Malondialdehyde
MDCK‐pHaMDR: Madin‐Darby canine kidney‐pancreatic heterogenous drug‐resistant cells
MeOH: Methanol
MFE:
*M. fragrans* Extract
MFEO/*β*‐CD:
*M. fragrans* Essential Oil / Beta‐Cyclodextrin Inclusion Complex MTA; White Mineral Trioxide Aggregate
NLMP: Nanoniosome‐Loaded *M. fragrans* Phenolic Compounds
NNE: Nutmeg Nut Extract
NO: Nitric Oxide
NSAIDs: Nonsteroidal Anti‐Inflammatory Drugs
OVCAR: Ovarian Carcinoma Cell Line
Papp: Apparent Permeability Coefficients
PC12: Pheochromocytoma 12
PCV: Packed Cell Volume
P‐gp: P‐glycoprotein
PRF: Platelet‐Rich Fibrin
PTZ: Pentylenetetrazole
SGOT: Serum Glutamate Oxaloacetate Transaminase
SGPT: Serum Glutamate Pyruvate Transaminase
SOD: Superoxide Dismutase
SREBP‐1c: sterol regulatory element‐binding protein 1c
STAT3: Signal Transducer and Activator of Transcription 3
TAA: Thioacetamide
TAC: Total Antioxidant Capacity
TLC: Thin‐Layer Chromatography
TNF‐R: Tumor Necrosis Factor Receptor

## 1 Introduction

Nature has been a primary source of healing and nourishment throughout human history. Among nature's vast offerings, plants provide food and are used as medicine for sustaining health and treating both noncommunicable and communicable diseases. (Salehi et al. 2020). Herbal medication, also known as plant‐based therapy, has a deep history in culture worldwide, including traditional Chinese medicine, ayurvedic medicine, folk medicine and African traditional medicine. These medicinal systems mainly used plants and their extracts to treat ailments and promote well‐being (Jamal 2023; Khalid et al. 2025). These traditional herbal medicines have established a strong reputation for effectiveness and minimal side effects (Chaughule and Barve 2024). Moreover, plant‐based bioactive compounds also offer various health benefits, thus making medicinal plants a promising candidate in the healthcare system (Sachdeva et al. 2020). These compounds positively impact physiological and metabolic functions, leading to immune‐ modulation and disease management (Embuscado 2019). Both developing and developed nations rely on these medicinal plants and their derived drugs for the management of various health anomalies such as hypertension (HTN), diabetes mellitus (DM), cancer, cardiovascular disorders (CVDs), gastro‐pulmonary issues, hepato‐renal syndrome (HRS), and neurogenerative diseases (Mehrotra 2021). The global herbal market is experiencing growth as consumers' awareness of these products increases. Major producers like India, China and the Middle East are dominating the market, with the prediction estimating that it will be reaching $5 trillion by the end of 2050, but deep research on their dosage and toxicity is needed along with the quality control (Hossain et al. 2022).

Nutmeg ( *Myristica fragrans* ), or Jaiphal, is a potent medicinal plant with a distinctive place that offers many health benefits and treatment for various health problems (Sultan et al. 2023). It is originated from Indonesia but now can be cultivated in other regions of the world, particularly in the rainforest regions of Taiwan, China, India, Malaysia, Sri Lanka, and South America (Sultan et al. 2023; Elfia and Susilo 2023). It is renowned due to its seed parts, having outer red aril (mace) and inner dark brown kernel (nutmeg) (Ashokkumar et al. 2022). The distinct flavor of this plant aids in utilizing it in many sweet and savory recipes and spices. The versatile and unique phytochemicals, such as phenols, terpenoids and essential oils (EO), exhibits therapeutic significance, such as antioxidant, anti‐inflammatory, anticancer (Tutuarima et al. 2024), antimalarial (Ibrahim et al. 2020), antifungal (Fernando and Senevirathne 2021), antibacterial (Okiki et al. 2023), antiplatelet (Arunachalam et al. 2018), antispasmodic (Prashant et al. 2013), anticonvulsant (Kumar and Samanta 2018), diuretics (Bhuiyan 2019), antidepressant (Dhingra and Sharma 2006), antidiabetic (Pashapoor et al. 2020), antihepatotoxic (Zhao et al. 2020), stomachic (Sattar et al. 2019), immune‐modulatory (El Shanawany et al. 2024), and analgesic (Bhuiyan 2019).

Nutritional profile, phytochemistry, medicinal properties, safety, and industrial applications of *M. fragrans* and its oil chemistry are the limelight of this review. In addition, the synergetic effect and comparative analyses of *M. fragrans* with the other plants or compounds in various health aspects are the highlight of this article. This review is unique and potent as the combination of health, safety, and economic views is guaranteed to be relevant in all disciplines.

## 2 Methodology

In this review article, a detailed literature framework has been utilized, which is dedicated to the therapeutic value of *M. fragrans*. Peer‐reviewed research work comprising phytochemical analyses and in vitro and in vivo regarding therapeutic potential was sourced from different high‐quality scientific databases such as PubMed, Google Scholar, Web of Science, Science Direct and Wiley Online Library. Keywords including nutmeg, *Myristica fragrans* , nutmeg, phytochemistry, bioactive compounds, myristicin, antioxidant, therapeutic potential, industrial application and toxicity were searched. Inclusion criteria included studies on *M. fragrans* nutritional and chemical profiling, health benefits, toxicity and applications. Studies with in vivo and in vitro analysis were included. Exclusion criteria: Studies other than *M. fragrans* and non‐English were excluded. These studies were evaluated to understand the dosage regimens, mechanism of action, physiological outcomes and potential toxicological responses.

### 2.1 Botanical and Morphological Depiction

**FIGURE 1:**
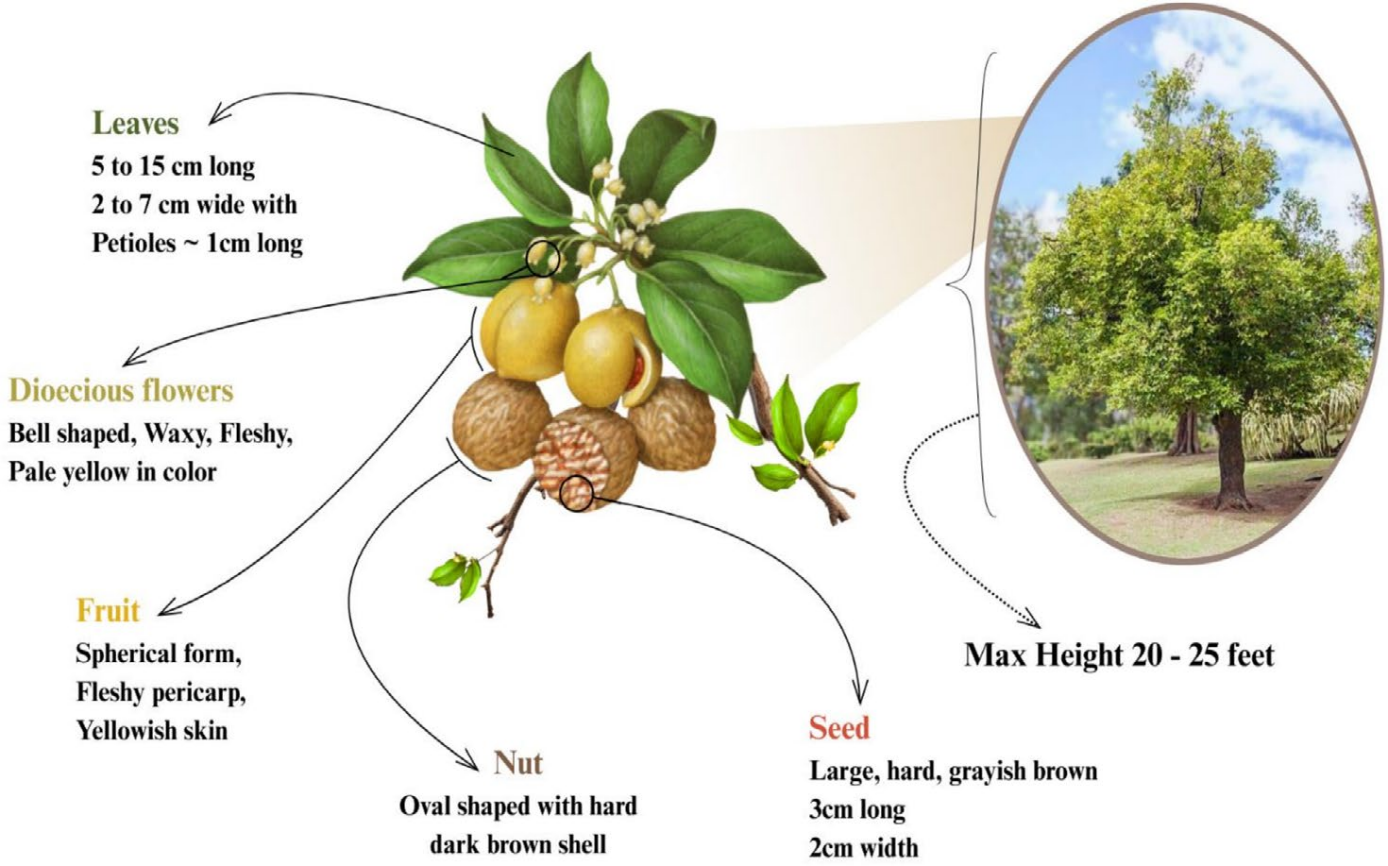
Botanical and morphological depiction of *Myristica fragrans*.

The tree of *M. fragrans* can reach a maximum height of 20‐25 feet with soft, grayish brown and spreading branches. Its barks usually have watery red or pink sap and gray‐brown stems. The dark green leaves are 5–5 cm long and 2–7 cm wide, with an elliptical shape along short, approximately 1 cm long petioles. Dioecious flowers are produced by trees that grow in small axillary racemes. These flowers are bell‐shaped, waxy, fleshy, and pale yellow. Female trees produce the succulent fruit, which splits into two at maturity. The fruit is spherical, with a fleshy pericarp and yellowish skin that splits to reveal a mace (a scarlet aril) surrounding the nut. The seed is large, complex, grayish brown and 3 cm long with a 2 cm width on average. The nut is broadly ovate or oval, encased in a rugged, dark brown shell, glossy outside and smooth inside. The tree bears this fruit throughout the year; April to November is the best time for harvesting (Ashokkumar et al. 2022; Ha et al. 2020). The foremost producers and exporters of *M. fragrans* are Indonesia and Granada. Other significant contributors are India, Malaysia, Sri Lanka, Papua New Guinea, and various Caribbean Islands (Gupta and Rajpurohit 2011). The botanical and morphological depiction of *M. fragrans* is demonstrated in Figure 1.

### 2.2 Common Names and Taxonomy

*M. fragrans* is also referred to as nutmeg in English. Nutmeg is derived from the Latin nux muscata, meaning musky nut. Worldwide, different common names, like Pala in Indonesian, Jaepatri in Bengali, Jafal in Kashmiri and Jaiphal in Hindi, Urdu, and Marathi, are assigned to it. *M. fragrans* belongs to the plantae kingdom, with the kingdom of Viridiplantae. It falls within the division Tracheophyta, which includes the vascular plants. It is further categorized under the class Magnoliopsida and the order Magnoliales. It is a member of the Myristicaceae family. The *Myristica* is the genus, and *Fragrans* is the species (Sultan et al. 2023).

### 2.3 Phytochemistry

**FIGURE 2:**
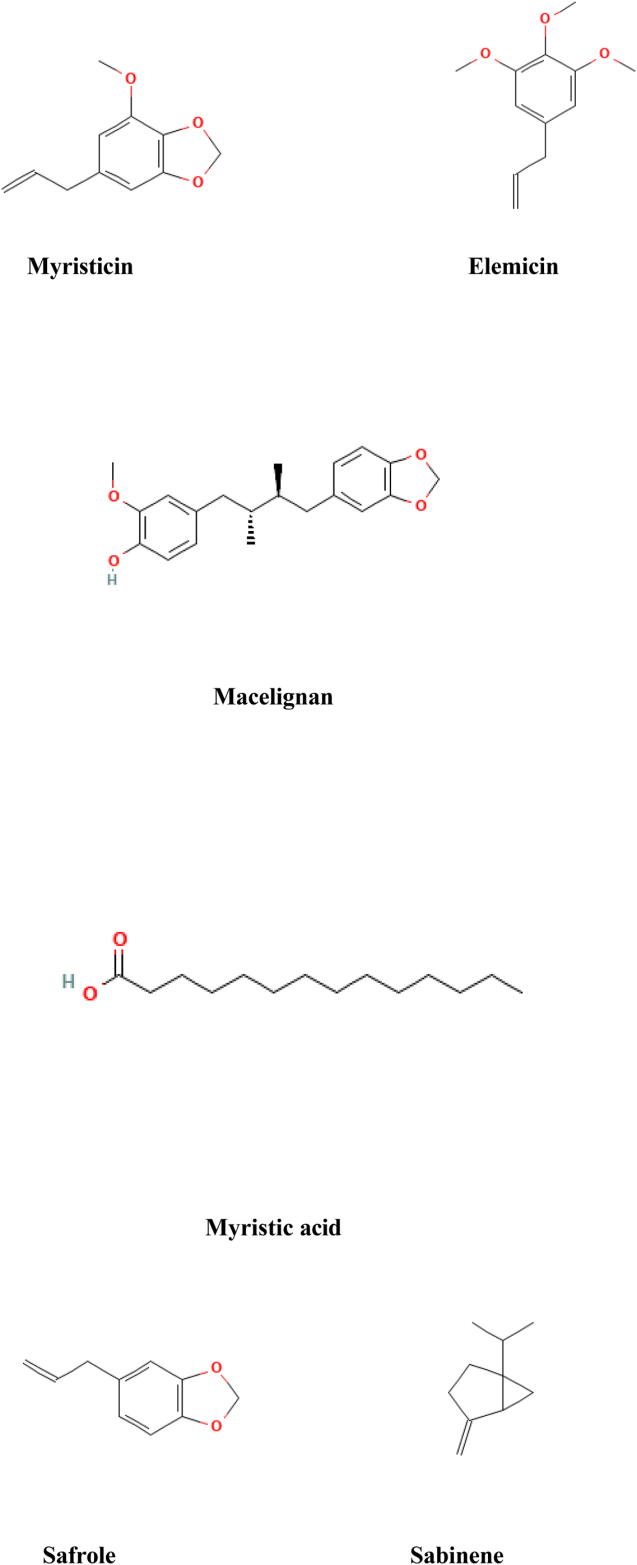
Phytochemicals of *Myristica fragrans*.

Phytochemicals are bioactive compounds derived from plant sources. These bioactive compounds give characteristics to fight against diseases and are found in whole grains, vegetables, fruits, herbs and nuts (Kumar et al. 2023). The phytochemicals of *M. fragrans* are displayed in Figure 2.

### 2.4 Seed

*M. fragrans* seed has bioactive compounds that give functional and therapeutic properties. The active components identified by GC‐MS analysis were myristicin, γ‐terpinene, isoborneol, and safrole (Okiki et al. 2023). Ghorbanian et al. (2019) found myristicin (11.17%), elemicin (22.16%), and myristic acid (39.93%) as the three main constituents of seed extract out of 17 isolated components. Compared to oval‐shaped extracts, the most significant quantities of myristicin were found in globose‐shaped seed and aril extracts, at 25.54 ± 2.37 mg/g and 10.60 ± 0.35 mg/g, respectively (Khamnuan et al. 2023). Furthermore, *M. fragrans* seed aqueous extract has more phenolic and flavonoid contents, alongside 8 bioactive compounds, as compared to the dichloromethane extract (Asika et al. 2016). Acetone (93.12 ± 1.48) was the most efficient solvent in extracting phenolic compounds, whereas the least was butanol extract (49.82 ± 1.26 mg GAE/100 g D.W) (Gupta et al. 2013). Furthermore, bioactive compounds, including flavonoids, phenols, alkaloids, terpenoids, tannin, saponin, steroids, diterpenes, glycosides, and anthraquinone, enhance the antioxidant profile of *M. fragrans* (Gupta et al. 2013; Olaleye et al. 2006; Sattar et al. 2019).

### 2.5 Pericarp

Pericarp, *M. fragrans* outer peel, contains beneficial compounds, that is, triterpenoids (0.06%), phenylpropanoids (0.28%), neolignans (0.13%), phenolic aldehydes (0.35%), steroids (0.49%), and sugars (10.20%) (Zhang et al. 2015), which enhance the free radical activity (Gupta et al. 2013; Rahman et al. 2018).

### 2.6 Fruit

Myristicin and safrol have been found in *M. fragrans* fruit. TLC‐Densitometry revealed myristicin content of 0.0017% (*w/w*) (Engel 2024). Moreover, 51 compounds, including 2‐pentadecanone‐like compounds, with myristicin (14.19%) were identified from chromatogram (Fernando and Senevirathne 2021).

### 2.7 Mace

The hydro‐methanolic extract of mace revealed the presence of phytochemicals, such as saponins (18%), phenolics (10%), tannins (16%), alkaloids (1%), and flavonoids (< 1%) (Ghorbanian et al. 2019; Ullah 2017). Moreover, chloroform fraction had the phenolic content of 127.18 ± 3.64 mg GAE/g dry fraction, however, the highest yield (25.39% ± 0.62% db) was observed by the methanol extract (Hasbullah et al. 2023).

### 2.8 Oil Chemistry

The EO extracted through hydro‐distillation yields 7.1%, characterized by a light‐yellow color with *M. fragrans* scent (Valente et al. 2011). Oil extracted from the leaves of *M. fragrans* gave 41 compounds, with a yield of only 1.20%. Almost 91% of its compounds were monoterpenes, and 66% of its content was *β*‐pinene, limonene, 4‐terpineol, α‐pinene and sabinene (Zachariah et al. 2008). A significant compound in its oil is sabinene, a monoterpene hydrocarbon, which accounts for 42.3% of EO in every 100g of *M. fragrans* (Nikolic et al. 2021). Other prominent compounds found in oil are myristicin and 4‐ terpineol (Muchtaridi et al. 2010). Myristicin contributes 12.94% of EO (Ansory et al. 2020). Flavonoids, alkaloids, tannins, terpenes, phenols, and glycosides were also identified by phytochemical examination of the extract, though saponins and coumarins were not found. Further analysis of oil shows that it also contains β‐pinene, α‐ pinene, cis‐sabinen hydrate, 4‐carene, isoelemicin, γ‐asarone, 2‐carene and α‐terpineol (with percentages 28.22%, 9.25%, 8.41%, 8.03%, 6.77%, 3.69%, 2.12%, and 1.22%, respectively) (Wang et al. 2019).

A comparison of the oil extracted from mace and seed indicated that monoterpene hydrocarbons and sesquiterpene hydrocarbons were isolated, and there was no discernible difference in the oil quality. The only slight variation was in amount. Monoterpene hydrocarbons, oxides, sesquiterpene hydrocarbons, aromatic chemicals, monoterpene alcohols, esters, and artificial fragrance compounds are all present in reasonable amounts in *M. fragrans* (Schenk and Lamparsky 1981). The results show that mace oil had a slightly higher yield of EO at 7.3%, whereas seed oil yielded 10.3%. The significant component found in mace was sabinene, while in the seed part were myristicin and safrole (Shafiq et al. 2016).

Interestingly, the myristicin content dramatically increased from 12.93% (in original oil) to 83.45% after distillation at different temperatures and pressure (Sudradjat et al. 2018). Soxhlet Extraction produced the highest oil yield with 75.69% of myristic acid in *M. fragrans* oil, with the primary sterol identified being β‐sitosterol (Obranović et al. 2020). Regarding extraction methods, ethyl acetate is the most complex with 51 components. With a percentage of 29.4%, sabinene was found to be the main constituent of the ethyl acetate oleoresins (Kapoor et al. 2013). The oleoresin produced using this UAE method has the most significant marks for the clove‐like feature, spicy and sweet scent, suggesting its outstanding quality, as well as a high ratio of β‐pinene to myristicin and safrole (Morsy 2016).

### 2.9 Nutritional Composition

*M. fragrans* seeds comprise of macronutrients and micronutrient alongside antinutrient compounds, such as phytates (0.38mg/100g) and tannins (0.15mg/100g) (Akinduko et al. 2024; Okiki et al. 2023). It has been found that the methanol seed extract has the calcium, magnesium, and iron content of 3.21 ± 0.05, 3.00 ± 0.05, and 1.00 ± 0.02 ppm, respectively. The atomic absorption spectroscopy revealed the presence of 4.59 ppm iron in the fruit (Syamani et al. 2011). Moreover, its seeds are enriched in essential amino acid, that is, leucine, valine, threonine, and non‐essential amino acids, that is, glutamate, aspartate, and arginine (Anaduaka et al. 2020). The proximate and mineral profile of *M. fragrans* seeds is presented in Tables 1 and 2.

**TABLE 1:** Proximate analysis of *M. fragrans* in different studies.

Proximate *analysis*	*M. fragrans* seeds	*M. fragrans* seeds	Aqueous extract
Moisture (%)	14.12	9.37 ± 0.06	13.78 ± 0.13
Protein (%)	7.02	18.67 ± 0.21	10.61 ± 2.10
Carbohydrate (%)	—	28.93 ± 0.61	22.43 ± 6.49
Fat (%)	25.60	26.73 ± 0.25	45.42 ± 1.15
Fiber (%)	13.80	12.43 ± 0.12	5.95 ± 0.19
Ash (%)	2.28	3.87 ± 0.21	1.81 ± 0.04
Energy (Kcal/kg)	—	3938.3 ± 2.08	—
References	(Asika et al. 2016)	(Okiki et al. 2023)	(Akinduko et al. 2024)

**TABLE 2:** Mineral content of *M. fragrans* in different studies.

Minerals	*M. fragrans* seeds (mg/kg)	*M. fragrans* seeds (mg/100 g)	Aqueous extract (ppm)
Fe^++^	—	7.63 ± 0.15	106.12 ± 0.95
Mg^++^	133.1	56.67 ± 2.98	403.63 ± 12.50
Ca^++^	1560.09	166.67 ± 7.64	161.55 ± 2.79
Zn^++^	—	0.53 ± 0.06	167.32 ± 5.81
PO_4_^−^	—	141.67 ± 7.64	—
Na	423.55	—	141.82 ± 1.07
K^+^	5139.00	68.33 ± 2.89	194.80 ± 3.63
References	(Asika et al. 2016)	(Okiki et al. 2023)	(Akinduko et al. 2024)

### 2.10 Bioavailability

Bioavailability is the rate of absorbed drugs or compounds into the bloodstream, which depends on administration route, signifying their safety and therapeutic role in improving disease management (Stielow et al. 2023). Studies exhibited that the mucous membrane of small intestine contributes their role in the absorption of *M. fragrans* . The hydrophilic and lipophilic compounds of *M. fragrans* enter bloodstream through oral route, which is subsequently distributed throughout the cells and organs of the body. The fatty diet improves the absorption of lipophilic compounds, like myristicin and elemicin in *M. fragrans* , and ultimately therapeutic profile (Al‐Rawi et al. 2024). Moreover, ~73% of ingested myristicin is excreted in urine as carbon dioxide within a day, which underscores the safety, efficacy, and detoxification mechanism of *M. fragrans* in the body.

The oral and intravenous administration of *M. fragrans* lignan depicted 16.2% and 100% bioavailability at 60 and 5 mg/kg body weight, respectively. Moreover, the mean residence time for oral administration was 10.02 h, demonstrating prolonged retention time. The absorbed *M. fragrans* into the bloodstream is transported to the target tissue and organ, where liver and intestine have been the examined tissues, having the highest concentration in tissue distribution studies (Song et al. 2019). The permeability of 8‐O‐4′‐type neolignans was found to be high, whereas benzofuran‐type neolignans showed medium to low permeability. The translocation and permeation of myrislignan and dehydrodiisoeugenol revealed time‐dependent response, suggesting high permeability and pharmaceutical aspects (Yang et al. 2010). Moreover, the permeability of lignans and phenolic malabaricones of *M. fragrans* seeds as a blood‐brain barrier (BBB) was determined via apparent permeability coefficients in MDCK‐pHaMDR cell monolayer model, which observed moderate‐to‐high BBB permeability of *M. fragrans* seeds. Additionally, 8‐O‐4′‐neolignans and tetrahydrofuran‐lignans, and low‐to‐moderate BBB permeability was detected with the benzo[furan]‐, dibenzylbutane‐ and arylnaphthalene‐type lignans. Malabaricones are poorly permeable and it has been observed that the chemical structures influence the BBB permeability and bioavailability (Wu et al. 2016).

### 2.11 Pharmacological Properties

**FIGURE 3:**
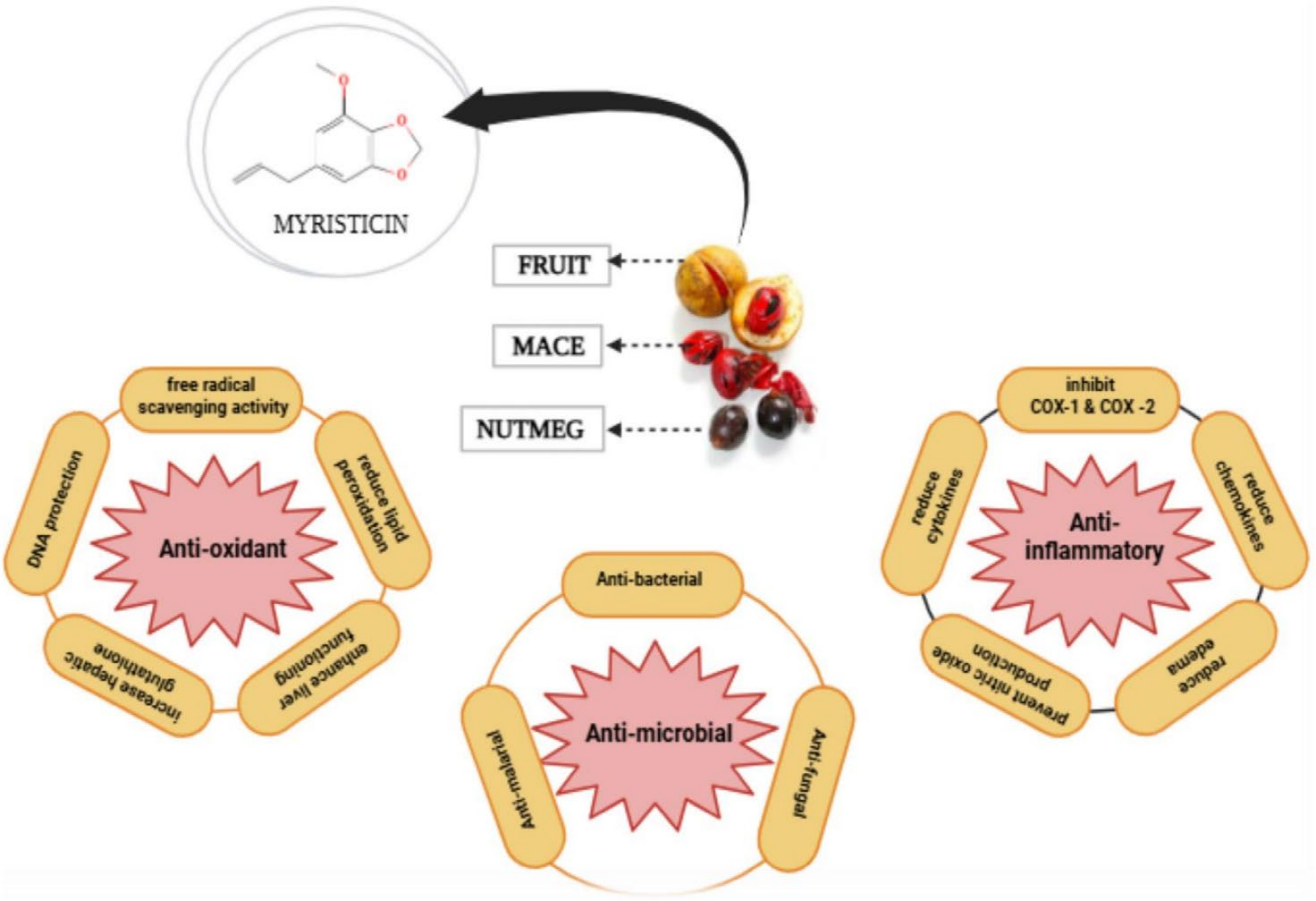
Antioxidant, antimicrobial, and anti‐inflammatory potential of ***Myristica fragrans*** by scavenging free radicals (ROS & RNS), DNA protection, reducing lipid peroxidation, inhibiting COX‐1 and COX‐2, decreasing nitric oxide production, inhibiting microbial growth, and suppressing cytokines and chemokines.

Medicinal plants contain a variety of pharmacological properties because of their rich bioactive compounds (alkaloids, flavonoids, terpenoids, glycosides, tannins, etc.). These natural elements have anti‐inflammatory, antimicrobial and antioxidant effects, among others. These substances have an effect on physiological systems, affecting enzymes, receptors and signaling pathways. These properties make medicinal plants important sources for drug discovery and traditional medicine all over the world (Alshaqhaa et al. 2025). Figure 3 provides information about the pharmacological properties of *M. fragrans* .

### 2.12 Antioxidant Potential

Free radicals in excess lead to damaging oxidative processes in the body that lead to a wide array of diseases. Antioxidants help plant species combat them and highlight their preventive and therapeutic phytotherapeutic role (Munteanu and Apetrei 2021). Many studies have shown the powerful antioxidant activity of *M. fragrans* . It was observed that both seed oil and its oleoresins have excellent antioxidant activity (Kapoor et al. 2013). Additionally, the major bioactive compound of EO, myristicin, has been demonstrated to exhibit a strong antioxidant activity that is even more effective than EO and its myristicin‐free counterpart. Thus, myristicin also isolated from *M. fragrans* protects against oxidative stress (Ansory et al. 2020). Furthermore, the concentration and incubation time‐dependent increase of the antioxidant activity of *M. fragrans* oil further demonstrated its potential as a natural antioxidant (Nikolic et al. 2021).

The free radical scavenging potential of *M. fragrans* was evaluated, which reported that acetone and butanol extract had maximum (63.04% ± 1.29%) and minimum (36.21 ± 1.31) DPPH activity (Gupta et al. 2013). Later, Assa et al. (2014) investigated the DPPH potential of *M. fragrans* , the results found highest DPPH radical scavenging (50%) and ferric ions reducing potential (82 mg GAE/g extract) of seed extracts. Furthermore, antioxidant indices were reported to be 0.6% and 44% for the respective reducing power and free radical scavenging activity of *M. fragrans* aqueous extract (Olaleye et al. 2006). Meanwhile, *M. fragrans* pericarp extracts (100 micrograms per milliliter) inhibited lipid peroxidation 82.5%–73.2%, thereby supporting antioxidant power (Zhang et al. 2015). β‐carotene‐linoleic acid assay showed that the *M. fragrans* mace extract was more effective in inhibiting lipid oxidation. DNA protection was observed with high doses (5 kg), which implies that *M. fragrans* mace extracts exhibit a strong free radical scavenging activity (Chatterjee et al. 2007). Table 3 shows the antioxidant activity of *M. fragrans* in different extracts.

**TABLE 3:** Antioxidant properties of *M. fragrans* different parts’ extracts in different studies.

Antioxidant		
Part/Extract	Findings	References
Methanol extract	Demonstrated strong antioxidant potential with IC50 = 2.56 ± 0.02 mg/L in DPPH assay	Hasbullah et al. 2023
n‐hexane extract	Exhibit strong antioxidant potential against MCF‐7 cell lines, with IC50 values of 99.76 ppm	Ginting 2021
Petroleum ether extract	DPPH assay depicted dose‐dependent scavenging potential with an IC50 value of 123.36 ± 0.76 μg/mL	Pashapoor et al. 2020
Methanol extract	DPPH assay revealed antioxidant activity by scavenging ~72% free radicals	Chakraborty et al. 2015
*M. fragrans* and its EO	↓ MDA levels, ↑ SOD, CAT, and GSH‐Px	Hassanen 2015
MeOH (95%) extract of seeds	Potent LDL‐antioxidant (IC50 = 2.6 μM) due to Dihydroguaiaretic acid lignin	Kwon et al. 2008
Alcoholic (50%) extract of seeds	↑hepatic GSH, ↓testicular LPO	Sharma and Kumar 2007

### 2.13 Antimicrobial Activity

Microbial infection is a significant threat to public health and antibiotic resistance in pathogens has emerged as an important public health problem in recent years (Pettinari et al. 2021). As a result, *M. fragrans* EO has attracted attention for its antimicrobial potential because of the presence of many terpene and aromatic components (Nikolic et al. 2021). Furthermore, as in MFEO/‐CD inclusion mixture, the increased solubility of water also increased the EO pathogen interaction region and antimicrobial activity (Wang et al. 2019).

Studies have revealed that *M. fragrans* has shown potential as an antibacterial, antifungal and antimalarial agent, though its findings are limited. Notably, macelignan at 5 mg/ml showed more decisive antibacterial action than EOs at 20 mg/ml. Additionally, *S. mutans* may be eradicated in about 1 min with 20 mg/ml of macelignan (Chung et al. 2006). *M. fragrans* EO has also shown potential as an antifungal. The composition of heated *M. fragrans* oleoresin changes but does not affect its antifungal efficacy. Sabinene is always the principal constituent after and before treatment, which gives it antifungal potential (Rodianawati et al. 2015). The antimicrobial activity of different parts and forms of *M. fragrans* is mentioned in Table 4.

**TABLE 4:** Antimicrobial activities of *M. fragrans* extracts and EOs.

Extract/part	Findings	Activity	References
Methanol extract of seed	↓ *C. accolens* , *P. acnes* , *S. aureus* , and *E. coli* , with 11–17 mm zone of inhibition at the concentration of 100 mg/mL	Antibacterial	Okiki et al. 2023
Methanol extract of seed	Active against *P. aeruginosa* , *E. coli* , *Salmonella species Serretia* sp. and *Klebsiella species*, demonstrating the inhibition zone ranged between 7 and 22 mm	Antibacterial	Chakraborty et al. 2015
Methanol extract	Produced a 2.2 cm inhibition zone against *E. coli*	Antibacterial	Varghese et al. 2017
Essential oil	*Bacillus subtilis* was highly sensitive to the extract (inhibition zone = 32 mm)	Antibacterial	Shafiq et al. 2016
Methanol extract	Showed a minimum bactericidal concentration of 7.8 mg/mL and a minimum inhibitory concentration of 3.9 mg/mL	Antibacterial	Chung et al. 2006
Essential oil	Showed better activity against *Fusarium oxysporum* (inhibition zone = 1.27 ± 0.18 cm) than *Aspergillus niger*	Antifungal	Fernando and Senevirathne 2021
Essential oil	Fungi at concentrations from 0.1% to 98%	Antifungal	Valente et al. 2011
Essential oil	Improved antifungal activity with zone of inhibition ranges from 18 to 45 mm	Antifungal	Shafiq et al. 2016
Essential oil	Mild activity against *Plasmodium falciparum* D6, with 39% inhibition at 1.0–2.5 min of distillation time	Antimalarial	Ibrahim et al. 2020

### 2.14 Anti‐Inflammatory Role

Inflammation, which engages adaptive and innate responses, is a typical reaction to infection. However, when it becomes uncontrolled, it can lead to autoimmune diseases, cancer or neurodegenerative diseases. Fortunately, many practical and safe anti‐inflammatory agents are available (Dinarello 2010). *M. fragrans* has proved its anti‐inflammatory potential via various mechanisms in different studies. Table 5 illuminates the anti‐inflammatory role of *M. fragrans* via possible mechanisms.

**TABLE 5:** Anti‐inflammatory role of *M. fragrans* in different studies.

Part	Findings	References
Ethanolic extract	↓LPS‐induced NO formation	Suthisamphat et al. 2020
Ethanolic extract	↓NO, cytokines, chemokines, and growth factors in dsRNA‐stimulated macrophages	Ghorbanian et al. 2019
Essential oil	P and COX‐2, thereby reducing edema and joint discomfort	Zhang et al. 2016
Hexane, ethyl acetate and methanolic extracts	↓COX‐1 and COX‐2 by 44%–42% and 47%–36%, respectively	Zhang et al. 2015
Ethanolic extract	Dose‐dependent reduction of inflammatory markers (IL‐6, IL‐1*β*, TNF‐α and NO), strongest at 50 μg/mL	Dewi et al. 2015
Chloroform extract	Reduced carrageenan‐induced paw edema in rats by 66% at 200 mg/kg dose	Olajide, Ajayi, et al. 2000
Essential oil	↓Carrageenan‐induced paw edema in rats with inhibition values of 75.0% (40 mg/kg) comparable to standard NSAIDs	Olajide, Ajayi, et al. 2000

### 2.15 Therapeutic Applications

**FIGURE 4:**
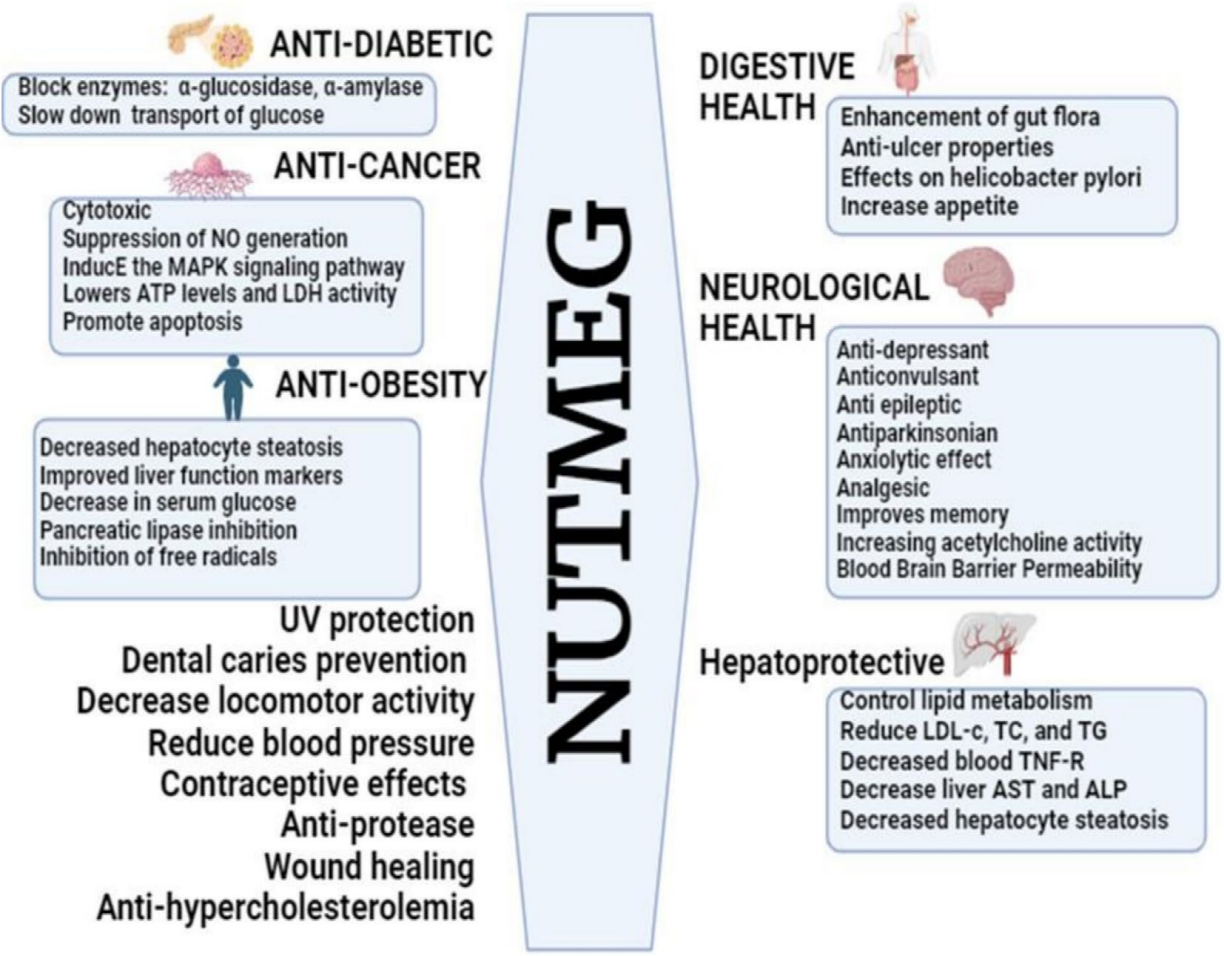
Therapeutic applications of *Myristica fragrans*.

Medicinal plants like *M. fragrans* possess vast therapeutic applications, offering natural remedies for various ailments. Used in traditional and modern medicine, they treat conditions like diabetes, hypertension, cancer, liver, renal and digestive disorders. Their bioactive compounds support healing, reduce side effects, and promote overall health and wellness. The therapeutic applications of *M. fragrans* are elaborated in Figure 4.

### 2.16 Neurological Health and CNS

Neurological disorders have become a major global health issue, with rising disability and mortality (Feigin et al. 2020). In low‐income countries, longer life expectancy results in more individuals reaching ages vulnerable to mental disorders, alongside widespread challenges such as poverty, illness, stress, unemployment, grief and substance abuse (Kenda et al. 2022). Anxiety and depression are common mental health problems affecting people globally. Between 2005 and 2015, epidemiological studies indicate that there was an 18.4% increase in the prevalence of depression. Also, a study revealed that in Pakistan, almost 47.7% of medical students experienced anxiety, highlighting a significant mental health concern for future and emphasizing the need to address it (Mirza et al. 2021). Natural products, such as phytochemicals of plants, have antioxidant and anti‐inflammatory potential that help prevent neurodegeneration and improve cognitive function (Kenda et al. 2022; Rehman et al. 2019). The bioactive compounds in *M. fragrans* are responsible for its neuropharmacological benefits on the CNS (Sultana et al. 2018). Its oil has demonstrated dose‐dependent anticonvulsant effect. In a study, its oil at 100 and 200 μL/kg prevented 62.5% and 50% of mice, respectively, against BIC‐induced clonic seizure (Wahab et al. 2009). Additionally, *M. fragrans* extract prevented pentylenetetrazol‐induced epileptic seizures, cell death, and glial activation (Ghorbanian et al. 2019; Kumar and Samanta 2018).

EO (100 nL/mL) increased GABA production due to the presence of α‐terpineol and myristicin, validating its conventional application in epilepsy management (Has et al. 2014). Moreover, 100 μg/mL *M. fragrans* extract suppressed (86.28%) H_2_O_2_‐induced oxidative injury in PC_12_ cells, revealing the neuroprotective effects. Malabaricone C exhibited the greatest activity among six isolated compounds against AChE and BChE with the highest IC50 value of 22.05 and 22.36 μM, respectively, and has the potential to treat diseases such as Alzheimer (Rastegari et al. 2022). *M. fragrans* is a potent enhancer of memory with memory‐enhancing and memory‐restoring actions (*p* < 0.001). Perhaps it is because it can reduce the acetylcholinesterase activity and raise the acetylcholine activity (Jissa et al. 2014). The 20 mg/kg ethanol extract of *M. fragrans* significantly improved motor function (*P* < 0.05), similar to the standard drug Pramipexole. So, extracts have antiparkinsonian potential (Palupi and Fekhayanti 2024).

The n‐hexane extract of *M. fragrans* has proved its potential as an antidepressant; the most effective results were shown at a 10 mg/kg dose (Dhingra and Sharma 2006). In a study, NNE is considered a safe natural treatment with antidepressant‐like qualities (Iwata et al. 2022). *M. fragrans* compound, 50‐methoxylicarin A, was most effective in inhibiting FAAH and interacting with the endocannabinoid system. *M. fragrans* has an impact as an anxiolytic without interfering with locomotor activity and was efficient at a 120 mg/kg dose (El‐Alfy et al. 2019). Also, it was found by ethanolic extract and ethyl acetate fraction that shortened the sleep latency and increased the sleep duration, thereby exhibiting sedative, anxiolytic, and analgesic effect (Mishra et al. 2018).

### 2.17 Antidiabetic Activity

DM is a metabolic disorder, characterized by hyperglycemia, leading to frequent urination, excessive thirst, and hunger (Kumar et al. 2020). It contributes to enhance health complications, such as stroke, renal injury, cardiovascular disorders, retinopathy, and neuropathy (Noman et al. 2025). The reduced insulin secretion and glucose sensitivity trigger the development of type 1 diabetes; however, insulin resistance worsens type 2 diabetes complications. Both conditions involve the progressive decline in *β*‐cell function, and early indicators like dysglycemia can improve early detection and risk prediction (Antar et al. 2023). The World Health Organization (WHO) supports incorporating medicinal plants in food to help treat diabetes mellitus (Yedjou et al. 2023).

Many studies have highlighted the growing focus on herbal plants and their phytochemical components as alternative treatments for diabetes (Tahrani et al. 2010). In a study, the hydroethanolic extract of seeds from *M. fragrans* (MFHE) produced silver nanoparticles, and these nanoparticles showed an intense antidiabetic action by blocking the enzymes like alpha‐ glucosidase and alpha‐amylase, along with lowering the transportation of glucose (Ramya and Krishnadhas 2021). While studying different extracts, it was found that the distilled water extract showed the most efficient α‐amylase inhibition. This shows the more substantial potential of mace as an antioxidant and antidiabetic than the acarbose and ascorbic acid (Hasbullah et al. 2023). The safrole‐free ethanol extracts also showed a drop in serum glucose and triglyceride levels in a type 2 diabetic rat model. The extract increased the PPAR *α* and γ activity. Therefore, it can treat dyslipidemia and diabetes (Lestari et al. 2019).

*M. fragrans* was compared with four other spices, that is, *Parmelia perlata*, *Illicium verum* , *Trachyspermum copticum* , and *M. malabarica* in streptozotocin‐induced diabetic rats. It was found that *M. fragrans* , *P. perlata* , and *M. malabarica* significantly reduce blood glucose and promote the dose‐dependent insulin secretion. Therefore, it was suggested that *M. fragrans* aids in diabetes management by multiple mechanisms, including enzyme inhibition and insulin secretion (Patil et al. 2011). When *M. fragrans* is compared or combined with a standard drug used to manage diabetes such as glimepiride, the treatment of diabetes and its related complication gives a good outcome. *M. fragrans* seeds n‐hexane, methanol, petroleum ether, and chloroform extract significant reduced blood glucose levels, that is, 60.7, 61.4, 63.6 and 65.1, respectively, in mice at 400 mg/kg body weight (Bhuiyan et al. 2018). In another experiment, Glimepiride and *M. fragrans* seed extract significantly lowered serum glucose of hyperglycemic mice when combined. Macelignan was found to have binding affinities with PPAR a and g of −9.2 and −8.3 kcal/mol. On the other hand, the affinity of glimepiride to the sulfonylurea receptor was −7.6 kcal/mol. It renders M. a good candidate in the treatment of diabetes mellitus (Nasreen et al. 2020). Comparing the extracts of *M. fragrans* and a standard drug, Jimmy and Effiong (2020) found that the extracts were equivalent to glibenclamide, and a long‐term application of aqueous extract of *M. fragrans* proved effective in the treatment of diabetes.

*M. fragrans* positively influences the pancreatic histology by enhancing the islet cells and pancreatic structure and decreasing necrosis. Greater doses such as 800 mg/kg can restructure the pancreas in diabetic rats (Ukwa et al. 2024). Oligosaccharides such as odoratisol A and dehydrodiisoeugenol contained in *M. fragrans* exhibit strong inhibitory activity against alpha‐glucosidase, implying their promising coagulation and diabetes control potential (Zhang, Tao, et al. 2016). In addition, one study indicates that methanol extract of the seed containing compounds such as malabaricone C and dehydrodiisoeugenol exhibits the greatest bioactivity, The antioxidant and anti‐alpha‐glucosidase effects of Malabaricone C emphasized the use of *M. fragrans* in the management of diabetes (Li et al. 2020). Moreover, malabaricone C and 3‐(3‐methyl‐5‐pentyl‐2‐furanyl)‐2(E)‐propenoic acid found in *M. fragrans* aril significantly alleviated α‐glucosidase and acetylcholinesterase activities, controlling postprandial hyperglycemia and oxidative stress (Sathya et al. 2020). Similarly, *M. fragrans* modulated GLUT4 translocation to cellular membrane by AMPK activation, and improves glucose uptake in muscle cells to reduce diabetes mellitus (Prabha et al. 2021).

**FIGURE 5:**
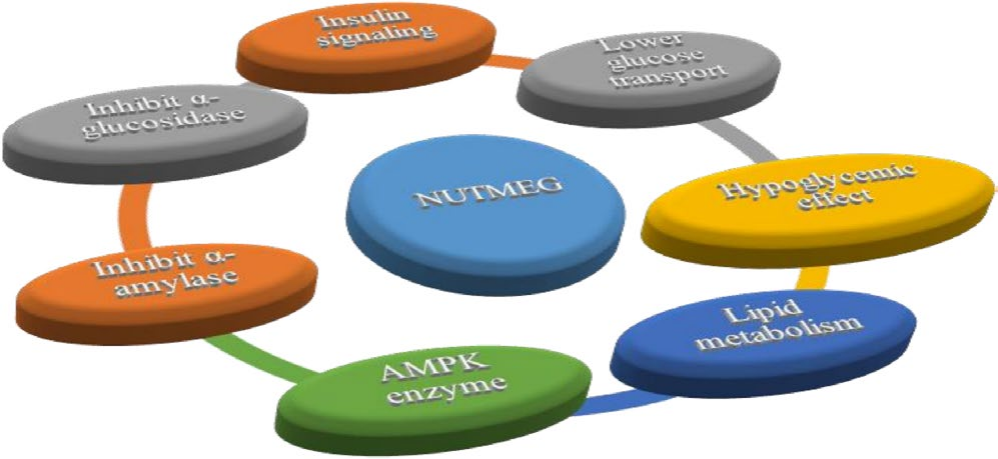
*Myristica fragrans* action for regulating diabetes mellitus.

The ethyl acetate seeds extract decreased fasting blood glucose levels and increased antioxidant enzymes to attenuate oxidative stress markers and glucose activities (Dey et al. 2018). Serum MDA levels increased markedly, although TAC levels decreased when using the dose of seed extract 100 and 200 mg/kg. *M. fragrans* may be a potential natural antioxidant for managing diabetes and its complications (Pashapoor et al. 2020). Figure 5 illustrates the antidiabetic activity of *M. fragrans* .

### 2.18 Anti‐Obesity

Obesity is a multifactorial condition where the buildup of excess body fat negatively impacts health (Lin and Li 2021). Obesity has become a critical public health issue, now ranked as the fifth leading cause of death worldwide. It results from an imbalance between calorie intake and expenditure, measured using BMI (Safaei et al. 2021). Obesity is characterized by inflammation and is associated with more than 50 health issues, including metabolic diseases, mental health disorders, joint problems, and several cancers. It also affects psychological well‐being and significantly strains global healthcare expenses (Westbury et al. 2023). Weight loss maintenance is the major challenge of obesity management (Perdomo et al. 2023).

It was found that oral AEN supplementation resulted in decreased hepatocyte steatosis and improved liver function markers, including ALT and AST. It lowered body weight and blood glucose levels in obese mice, eventually helping to treat obesity (Zhao et al. 2020). On assessment, the renal parameters and functions remained consistent by extracts of *M. fragrans* , but a decrease in serum glucose, body weight and cholesterol, and liver enzymes like SGOT, ALP and SGPT. *M. fragrans* is helpful in the treatment of obesity and hyperlipidemia by optimizing the activity of the AMKP and pancreatic lipase enzymes (Vangoori et al. 2019a).

Oral intake of ethanolic extract of *M. fragrans* was found to be effective in reducing body composition particularly the body weight, BMI, total fat mass, adipose tissue and organ weights of the cafeteria diet‐induced obese rats. This was extremely evident at 400 mg/kg, compared to 200 mg/kg doses (Yakaiah et al. 2019). In the course of carrying out a study, it was found that *M. fragrans* ethanolic extract exhibits potential as an antioxidant because at a solution concentration of 5 mg/mL, 88% of DPPH radicals are inhibited, just like ascorbic acid. In addition, the highest pancreatic lipase inhibition (66.24) was recorded at 100 ug/mL. Therefore, *M. fragrans* ethanolic extract had great potential as an alternative obesity treatment (Vangoori et al. 2019b).

### 2.19 Anticancer Properties

Cancer or uncontrolled cell growth develops when an imbalance between cell apoptosis and proliferation occurs. The intrinsic apoptotic pathway influences the mitochondrial membrane to suppress Bcl‐2 and Bcl‐xL protein expression (Kabir et al. 2021). According to GLOBOCAN, globally, there were approximately 9.7 million deaths from cancer and an estimated 20 million new cases in 2022 (Bray et al. 2024). Cancer is the second most common cause of death worldwide, with lung, breast and colorectal cancers having the highest rates of occurrence and mortality. While advancements in prevention, early detection, and treatment have reduced cancer deaths, risk factors such as smoking, poor diet and lack of exercise remain major contributors (Maaz et al. 2025). Persistent inflammation fosters tumor development by promoting cell proliferation, survival, and immune dysregulation, contributing to cancer progression and resistance to therapy (Hou et al. 2021).

The increasing interest in cancer treatment has led researchers to explore plants as sources of bioactive compounds with potential anticancer, antioxidant, anti‐inflammatory, and antimicrobial effects (Garcia‐Oliveira et al. 2021). In several studies, *M. fragrans* has anticancer effects due to the modulation of several signaling pathways (PI3K/Akt/mTOR, MAPK, and NF‐κB) and the G0/G1 or G2/M phase arrest, mostly attributed to the presence of bioactive constituents, including myristicin (Ha et al. 2020). A research study reported that the ethanol extracts are cytotoxic since the extracts can inhibit LPS‐induced NO release with IC50 values of 26.06 and 82.19 ug/ml, respectively. The ethanolic extract had much better cytotoxic potential than the water extract (Suthisamphat et al. 2020). Moreover, the ethanolic extract of *M. fragrans* expressed over 70 percent growth inhibition against all human cancer cell lines. OVCAR‐5 demonstrated the extract's maximal cytotoxicity of 97%, with 86% of two human cancer cell lines, Colon 5,02,713 and PC‐5, being active. Thus, the plant has a potential anticancer action (Prakash and Gupta 2013). Additionally, *M. fragrans* seed's ethanolic extract and 10 μg/mL of pure quercetin were the least cytotoxic (Dewi et al. 2015).

The methanolic extracts from the globose‐shaped *M. fragrans* arils demonstrated a 37.26% suppression of NO generation in RAW 264.7 cells (Khamnuan et al. 2023). In a study, methanol extract showed modest anticancer activity against the MG‐63 cell line, 63% cell suppression and 40.39 μg/ml IC50 at a dose of 50 μg/ml (Chakraborty et al. 2015). DHGA showed potent cytotoxic activities against most cancer cells, with IC50 values highest for Hela, 30.0 μM and lowest for H358, 10.1 μM. Furthermore, three other lignans with anticancer properties include macelignan, fragransin A2, and nectrandrin B (Thuong et al. 2014). With an IC50 value of 12.3 μM, Malabaricone C, whose chemical structure is a dihydroxyphenyl nonanone skeleton, had the inhibitor action against the A549 cancer cell line (Cuong et al. 2012). Furthermore, C18H30O4, an isolated chemical, has strong anticancer effects against MCF‐7 cell lines, with IC50 values of 10.75 ppm (Ginting 2021). By inducing the MAPK signaling pathway and enhancing the production of cleaved caspase‐3 and poly‐ADP ribose polymerase, Myrifragranone C from *M. fragrans* also caused apoptosis in cells and increased its potential as an anticancer drug (Phong et al. 2023). In normoxia and hypoxia, *M. fragrans* treatment decreases the ATP level, LDH activity and lactate production in cancer cells, and consequently, reduces the metabolic rate. Besides, the in vivo test through mouse model on tumor growth revealed that *M. fragrans* could effectively inhibit tumor growth in lung carcinoma cells as well (Kim et al. 2016). The expression and cell viability of Bcl‐2 are reduced by Mace extract, which inhibits the proliferation of cancer cells, and induces apoptosis. Hence, mace extract is important as an oral cancer chemopreventive agent (Rengasamy et al. 2018). Similar to cisplatin, the ethyl acetate fraction of *M. fragrans* also greatly decreased the proliferation of B16‐F10 cells and triggered caspase‐3 to induce apoptosis. This means that it has high potential as chemopreventive of skin cancer melanoma (Susianti et al. 2021).

### 2.20 Gastrointestinal Benefits

The gastrointestinal tract is the part of the body that processes food, absorbs nutrients, and gets rid of waste, and all these processes are coordinated by different tissues and cells (Liao et al. 2009). The gastrointestinal system is a very important part of the entire health of a person and gut maturation has been shown to be affected by weaning, corticosteroids and some phytochemicals. Research suggests that medicinal plants and their compounds may support gut development (Mukonowenzou et al. 2021). It was demonstrated that MFE increased the strength of gut flora and boosted the number of probiotics. This improvement in gut microbiota contributes to overall digestive health and significantly prevents GI disorders (Zhao et al. 2022). In addition to their potent antioxidant properties, flavonoids have properties to fight against ulcers and inflammation. In a study, at 200 mg/kg, *M. fragrans* significantly (*P* < 0.05) decreased stomach lesions by 40.98% in models of ethanol‐induced ulcers (Sattar et al. 2019).

*Helicobacter pylori* can cause severe gastric disorders (Chuah et al. 2011). Research indicates that compared to water extract, ethanolic extract exhibits significantly greater anti‐*H pylori*, cytotoxic and anti‐inflammatory properties. Thus, mace extract can treat gastrointestinal disorders (Suthisamphat et al. 2020). However, it should be consumed in a safe dosage range, as *M. fragrans* at high dose, like 20 g, causes severe tissue deterioration and elevated serum glucose levels. At the same time, normal stomach histology was observed at low dose in rats (Mbadugha et al. 2019). This highlights the necessity for careful dosage management when using *M. fragrans* for therapeutic purposes. Furthermore, it was observed that compounds like phenylpropanoids in the oil dramatically increased meal intake. Not like benzylacetone, which increases the weight, continuous exposure to methyl eugenol causes olfactory habituation, making plant use in increasing appetite (Ogawa and Ito 2019).

### 2.21 Hepatoprotective Role

The liver is a vital organ responsible for metabolism, detoxification, nutrient storage, and maintaining bodily balance. Its function in filtering substances from the bloodstream makes it prone to damage, leading to liver dysfunction (Adewusi and Afolayan 2010). Natural active compounds are increasingly recognized for their low toxicity and numerous health benefits, including preventing obesity, diabetes, and metabolic syndrome, with key phytochemicals for these effects (Yao et al. 2023). Some studies prove *M. fragrans* potential as hepatoprotective, such as in a study, it was demonstrated that MFE enhanced liver function, reduced fasting blood glucose levels, and successfully reduced serum lipid levels (LDL‐c, TC, and TG). In mice given a high‐fat diet, *M. fragrans* extract dramatically controls inflammation and lipid metabolism (Zhao et al. 2022).

*M. fragrans* seed had the highest hepatoprotective effect when 500 grams of the powder were extracted consecutively with n‐hexane, ethyl acetate, and 70% ethanol. The main ingredient of *M. fragrans* , myristicin, dramatically decreased blood TNF‐R concentrations in the liver‐damaged rats (Morita et al. 2003). Serum levels of AST, ALT, ALP, total bilirubin, and albumin increased significantly in the treated groups by *M. fragrans* , but liver AST and ALP activity, total protein, and albumin decreased (Akinduko et al. 2024). In a study, restoring normal lysophosphatidylcholine metabolism, decreasing ALT and AST levels, and lowering oxidative stress, NME guards against TAA‐induced liver damage. The key ingredient in *M. fragrans* is myrislignan, which contributes significantly to the protective effect (Yang et al. 2018).

It was also observed that AEN therapy successfully decreased the expression of genes linked to lipid production, including *SREBP‐1c* and *FASN*, decreasing the amount of lipid in cells. Furthermore, oral AEN supplementation decreased hepatocyte steatosis and improved liver function markers, including ALT and AST (Zhao et al. 2020). During research, all groups of rats gained considerable weight, although their relative liver weight did not alter much by consumption of 250‐750 mg/kg *M. fragrans* powder (Aladeyelu and Oyewo 2018). NLMP treatment causes significant reductions in liver enzymes, triglycerides, cholesterol, and total bilirubin levels, as well as improvements in weight and feed intake in mice with liver toxicity. Furthermore, NLMP raised antioxidant enzyme levels in the liver and lowered the expression of inflammatory genes (Poorbagher et al. 2022).

### 2.22 Miscellaneous

The miscellaneous health benefits of *M. fragrans* in different studies are stated in Table 6.

**TABLE 6:** Miscellaneous benefits of *M. fragrans* in different studies.

Finding	Activity	References
↓Lipid accumulation by inhibiting FAS and STAT3	Antifat accumulation	Perumal et al. 2023
Promoted oral wound healing without any adverse effect	Wound healing	Deepthi et al. 2023
Effective pulpotomy substitute in primary teeth, like	Pulpotomy	Setty et al. 2022
MTA	substitute	—
↓Ovarian follicle and endometrial gland formation; affecting weight in reproductive stage	Contraceptive	Sakpa and Eguavoen 2021
↓Blood pressure in hypertensive elderly patients	Anti hypertensive	Nugraha et al. 2020
EO (92% myristicin) showed strong UV protection	Sunscreen	Ansory et al. 2020
Ethanolic extracts enhanced PRF preservation	Antiprotease	Arunachalam et al. 2018
↓ Symptoms and more effective with continuous use	Morphine addiction treatment	Zaheer et al. 2016
↓ Harmful effects of hypercholesterolemia	Hepato protective	Hassanen 2015
Low dose showed positive impact on testicular germinal epithelial, spleen, and liver	Anti hyperlipidemic cytotoxicity	Moheb et al. 2014
↓ Locomotor activity	Sedative	Muchtaridi et al. 2010
Effective substitute in dental caries prevention; candidate for oral care products	Dental care	Chung et al. 2006
Extract (500 mg/kg) ↑ sexual performance metrics, ↓ latencies and intervals	Aphrodisiac	Tajuddin et al. 2005
↓ Fever, pain, diarrhea, and inflammation	Antipyretic Analgesic	Olajide, Ajayi, et al. 2000
Protected against ADP/adrenaline‐induced thrombosis, ↓ carrageenan‐induced paw edema	Antithrombosis	Olajide, Makinde, and Awe 2000

### 2.23 Adverse Effects and Toxicity

As medicinal plants' popularity increases due to their perceived safety and affordability compared to conventional medicines, there are reports of toxicity and allergic reactions (Faisal et al. 2019). Table 7 includes safety and toxicity studies of *M. fragrans* in different forms.

**TABLE 7:** Studies showing toxic effects of *M. fragrans* .

Plant part	Dose	Duration	Findings	References
Aqueous Extract	400 mg/kg	28 days	↓ PCV, Hb, MCV, platelets	Akinduko et al. 2024
Methanol and n‐hexane Extracts	1000 mg/kg	7–14 days	↑ Urea, bilirubin, creatinine, liver enzymes; liver and kidney damage risk with prolonged high‐dose use	Anaduaka et al. 2022
Powder in normal saline	4.0 g/kg seed in 0.2 mL Normal saline	7–14 days	↓ Hepatic structure clarity and growth,↑ ALT, central vein and portal vein dilation	Cao et al. 2020
Seed	750 mg/kg	4 weeks	Caused minor sinusoidal dilation and modest, ↑ hepatocytes near central vein	Aladeyelu and Oyewo 2018
Ethanolic extract	500 mg/kg,	14 days	↓Hemopoietic activity, possibly due to Saponin‐induced cell lysis	Bamidele et al. 2011
Seed	1 and 2 g\kg\day	32 days	Caused stomach epithelial hyperplasia, atrophic degeneration, and possible ulcerative/neoplastic changes	Adjene and Igbigbi 2010
Seed	1000 mg/kg\day	42 days	Significant renal damage (cytoarchitectural deformation, ↓renal corpuscles, ↑tubular epithelial cell death)	Eweka and Eweka 2010
Oil (dried kernel extract)	600 μL/kg	5, 30, 60, and 120 min	Did not affect mice's ability to climb inverted screen	Wahab et al. 2009
	LD50 = 2150 μL/kg	24 h	Righting reflex loss, seizures, clonus	
	HD50 = 1265 μL/kg	10–60 min	Righting reflex lost within 10 min, recovered in 60 min	
Aqueous extract	400–500 mg/kg	28 days	↑Degradation of testis germinal epithelial cells and spleen alteration (lymphoid depletion, necrosis)	Olaleye et al. 2006
Kernel	—	—	Contained *Lasiodipia theobromae* (50%) and aflatoxin‐producing fungi, posing contamination and health risks	Arifah et al. 2022

### 2.24 Industrial Application

Recent studies have shown that *M. fragrans* also played a role in the cosmetic industry. Its EO is commonly incorporated into shaving creams, shampoos, perfumes, soaps, etc. It can also be utilized in the formulation of medicinal syrups and balms. Due to the ability of mace lignin to inhibit melanin production, it possesses skin whitening properties (Naeem et al. 2016). *M. fragrans* oil is used in men's fragrances and toiletries for its distinctive aromatic qualities (Rema and Krishnamoorthy 2012). *M. fragrans* is utilized in different products such as pickles, cookies, preserves, powders and chutneys. Ground form of seed is commonly preferred in the food industry. As a grounded form can lose its flavor quickly, it is grated fresh before baking and frying. It pairs well with bananas, vanilla and chocolate, adding a spicy, sweet aroma to baked goods like pumpkin pies and pastries (Malik et al. 2022). Its warm, sweet flavor and scent enhance the production of, including dairy items, beverages, spice mixes, and meats. It is also incorporated into chewing gum, teas, candies, soft drinks and also blended with alcohol or milk (Periasamy et al. 2016).

Pinene in *M. fragrans* oil produces solvents, plasticizers, camphor and synthetic pine oil. Dipentene is utilized in making resins and as a dispersing agent. Myristic acid is a key ingredient in plastics, perfumes, shampoo, soaps, paints, greases, rubber compounding and food grade additives (Rema and Krishnamoorthy 2012). *M. fragrans* and its oleoresin are used extensively in cosmetics, soap production and pharmaceutical. Also, it is a major participant in the food business. The safety and aflatoxin‐free nature of *M. fragrans‐*based products, oleoresin and oils enhance its importance in food production. (Al‐Rawi et al. 2024).

## 3 Limitations, Conclusion, and Future Perspective

The bioactive compounds of *M. fragrans* augment its therapeutic, medicinal, and industrial application. Compounds, like myristicin, eugenol, macelignan, and sabrole, reduce oxidative stress, regulate immune response, and reveal protective effects against multiple health complications. Furthermore, the traditional and conventional medicinal practices in diverse cultures validate its importance as a therapeutic agent. *M. fragrans* exhibited anti‐inflammatory, antimicrobial, antioxidant, and anticancer effects by alleviating oxidative stress and inflammatory markers. Moreover, *M. fragrans* and its derivatives are widely exploited in medicinal, cosmetics, and food industries.

Although various research has been conducted, future research should focus on isolating and characterizing the other bioactive constituents and illuminating the mechanism of action of these constituents. Well‐designed clinical trials and long‐term safety assessment are required to validate its health‐promoting attributes and facilitate its incorporation into evidence‐based therapeutics. Moreover, stability and bioavailability of bioactive compounds are significant challenges. Therefore, there is also potential to explore the delivery systems to enhance the bioavailability of key components. Additionally, integrating plants into nutraceutical and herbal formulations could provide a synergistic approach to managing disorders related to the modern lifestyle. In summary, *M. fragrans* presents a significant addition to medicinal and dietary practices, with its bioactive compounds offering various health benefits. Continued exploration of its therapeutic properties may pave the way for new natural products to enhance health and wellness.

## Ethics Statement

The authors have nothing to report.

## Consent

All authors are willing to publish this manuscript.

## Conflicts of Interest

The authors declare no conflicts of interest.

## Data Availability

The data that support the findings of this study are available from the corresponding author upon reasonable request.
